# Non-Surgical Ear Rejuvenation Through Selective Neuromodulation with Onabotulinum and Abobotulinum Toxin-A

**DOI:** 10.3390/toxins18090366

**Published:** 2026-08-26

**Authors:** Paola Rosalba Russo, Andrea Sbarbati, Emanuele Bartoletti, Loredana Cavalieri, Maurizio Cavallini, Valentina Pinto, Giovanni Salti, Sheila Veronese

**Affiliations:** 1 Estemed Clinic, 41122 Modena, Italy; pol.estemed@gmail.com; 2 Department of Neuroscience, Biomedicine and Movement Sciences, University of Verona, 37134 Verona, Italy; andrea.sbarbati@univr.it; 3 Department of Aesthetic Medicine, Gemelli Isola Tiberina Hospital, 00186 Rome, Italy; ebartoletti@lamedicinaestetica.it; 4 Plastic Reconstructive and Aesthetic Surgery Unit, Italian Society of Aesthetic Medicine, 00195 Rome, Italy; cavalieri.loredana@libero.it; 5 Clinical and Educational Center Agorà, 20122 Milan, Italy; maurizio.cavallini@libero.it; 6 Department of Plastic and Reconstructive Surgery, Modena University Hospital, 41124 Modena, Italy; valentina.pintomd@gmail.com; 7 Medlight Institute, 50144 Florence, Italy; info@giovannisalti.com

**Keywords:** botulinum toxin type A, ear aging, auricle aging, auricle aging classification, auricle aging treatment

## Abstract

To date, there are no studies concerning the ear aging phases, nor adequate anti-aging protocols. The aims of this study are: (1) to propose an aesthetic classification of auricular aging; (2) to evaluate the aesthetic implications, efficacy, and safety levels of botulinum toxin application to the auricles as anti-aging treatment. A retrospective study was conducted in a single center on 40 patients’ charts to define an aesthetic ear aging classification, focusing on the muscles involved in the aging process; then an analysis on the results of the treatment of 50 healthy women (50–65 years), with the botulinum toxin type A for ear rejuvenation was conducted; the technique, visual aesthetic results, incidence of side effects, and efficiency of the treatment were presented. All procedures were performed in 2024, and subjects were monitored for the duration of the effects. Auricle aging process may be classified into 4 degrees. The treatment of 50 women with aging ears degree 1–2 permits correction of both ear protrusion and shape. In 1.5% of the auricles, mild swelling and transient bruising at the injection site were observed immediately after the treatment and resolved within 2–3 days. Patient satisfaction was high, and two external reviewers confirmed the treatment’s aesthetic efficiency. Aesthetic classification of auricle aging can be a useful tool in anti-aging decision-making. Ear treatment with botulinum toxin type A seems complementary to face treatment. The results are encouraging, but the number of subjects treated is limited. Further studies are required to outline age-related guidelines.

## 1. Introduction

Ears are part of the face and, like other facial parts, can present imperfections related to muscle hypertonicity, skin sagging, or congenital conditions [1,2,3]. Auricular aesthetics contributes significantly to facial harmony and can give rise to psychological issues [4,5]. Moreover, the auricles and the mandible originate from the first and second pharyngeal arches; consequently, auricular imperfections are often associated with facial asymmetry [6], which can cause psychological, emotional, and physiological distress [6]. Correction of facial asymmetry has been shown to improve psychosocial outcomes [7].

Traditionally, auricle abnormalities, including protruding ears, are corrected surgically [8,9,10,11,12]. Although surgery provides permanent structural corrections, it requires anesthesia and may not always match changing aesthetic needs over time [10,13,14,15].

Ear aging involves anatomical and physiological changes, particularly affecting cartilage [16,17,18,19,20]. Reduced cartilage cell size, glycosaminoglycans, and elastin contribute to decreased connective tissue strength and lobe elongation, while gravitational effects may further influence ear morphology [21]. Although auricle features vary among individuals due to genetic factors [22,23], common age-related changes include:

- Skin laxity—caused by a reduction in collagen and elasticity, it determines a decrease in skin tone and the appearance of wrinkles and pronounced folds [24];
- Thinning of the earlobe—caused by a volume loss, it determines the elongated shape of the earlobe [18];
- Muscle overactivity—it can accentuate wrinkles and skin folds around the auricle [25];
- Loss of cartilage definition—it causes cartilage remodeling correlated to a loss of elasticity, contributing to a less defined appearance of the auricle [26];
- Increased ear protrusion—observed in some individuals, it is an increase in ear protrusion caused by relaxation of the soft tissues and cartilage [18];
- Skin pigmentation alterations—they may include age spots, actinic keratoses, or non-melanoma skin cancers [27].

Overall, auricular aging involves a combination of structural, volumetric, and cutaneous changes that affect multiple anatomical components of the auricle, including the skin, soft tissues, and cartilage. Together, these modifications progressively alter ear shape and contour, contributing to a less defined and more aged appearance.

To address facial aging, non-invasive or minimally invasive procedures have increasingly been adopted [28,29]. Among these, hyaluronic acid (HA) fillers [30,31] and botulinum toxin (BoNT) [32,33,34,35,36,37,38] are widely used, including in several off-label applications [39,40,41,42]. The choice of the type of intervention depends on the severity of the defect to be corrected. For earlobe sagging, for instance, the Mowlavi’s scale is used to define the treatment [43]. The scale classifies earlobe ptosis into five degrees (I-V) based on a survey in North American Caucasians, which established the preferred otobasion inferius-to-subaurale distances in male and female faces. According to this scale [44], when the ptosis grade is ≥II (6–10 mm), surgery is required [45]; when ptosis is <II, fillers are used to volumize the earlobe and correct helix anomalies [46,47,48,49], and their effects last 6 to 12 months, depending on the product type [50].

Although the number of ear rejuvenation procedures is not routinely recorded worldwide [51], cosmetic ear surgery accounted for 2.1% of the total cosmetic procedures globally, and increased by 42.7% between 2020 and 2024 and by 3.8% between 2023 and 2024, indicating growing interest in auricular aesthetics [52]. Correction procedures have demonstrated benefits for quality of life and psychological well-being in both children and adults [53,54].

Despite the increasing demand for ear aesthetic treatments and the widespread use of BoNT in facial rejuvenation, no evidence is currently available regarding its use for auricular correction or the treatment of age-related auricular blemishes. Therefore, the aims of this study are: (1) to propose an aesthetic classification of auricular aging, helpful in facilitating the choice of therapeutic treatments; (2) to evaluate the aesthetic implications, efficacy, and safety levels of BoNT application to the auricles as a potential anti-aging treatment.

## 2. Results

### 2.1. Aesthetic Ear Aging Classification

**Figure 1:**
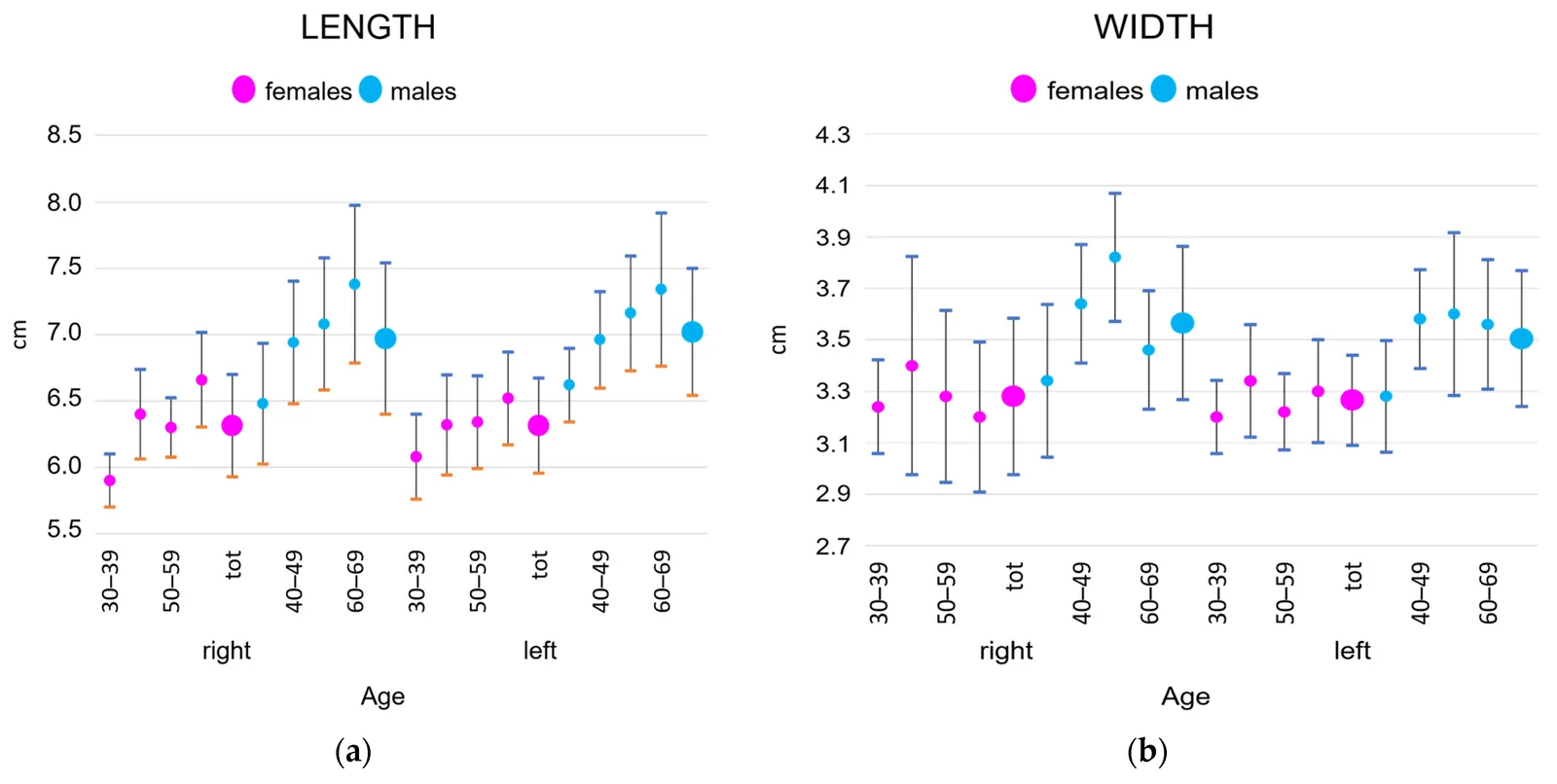
Measures of the cohort of 40 subjects considered to define the aging pattern. (**a**) The length of the auricles increases over time for both females and males. A marked gender difference is evident, with males presenting longer auricles than females at all ages; (**b**) the width of the auricles changes over time for both females and males, but there is not a clear pattern of change. As for length, a marked gender difference is evident, with males presenting wider auricles than females after the age of 40. In the 30–39 age range, the differences are minimal.

The measures of the mean and standard deviations of the auricles of the cohort of 40 subjects considered in this study are represented in Figure 1. Both for females and males, a progressive increase in the length of the auricles was observed, while the width was stable. Consequently, the auricular index decreased over time. Males, generally, presented auricles longer and larger than those of females.

The four degrees of aesthetic aging, defined according to the aging patterns observed in the 40 patients studied, are detailed in Table 1.

**Figure 2:**
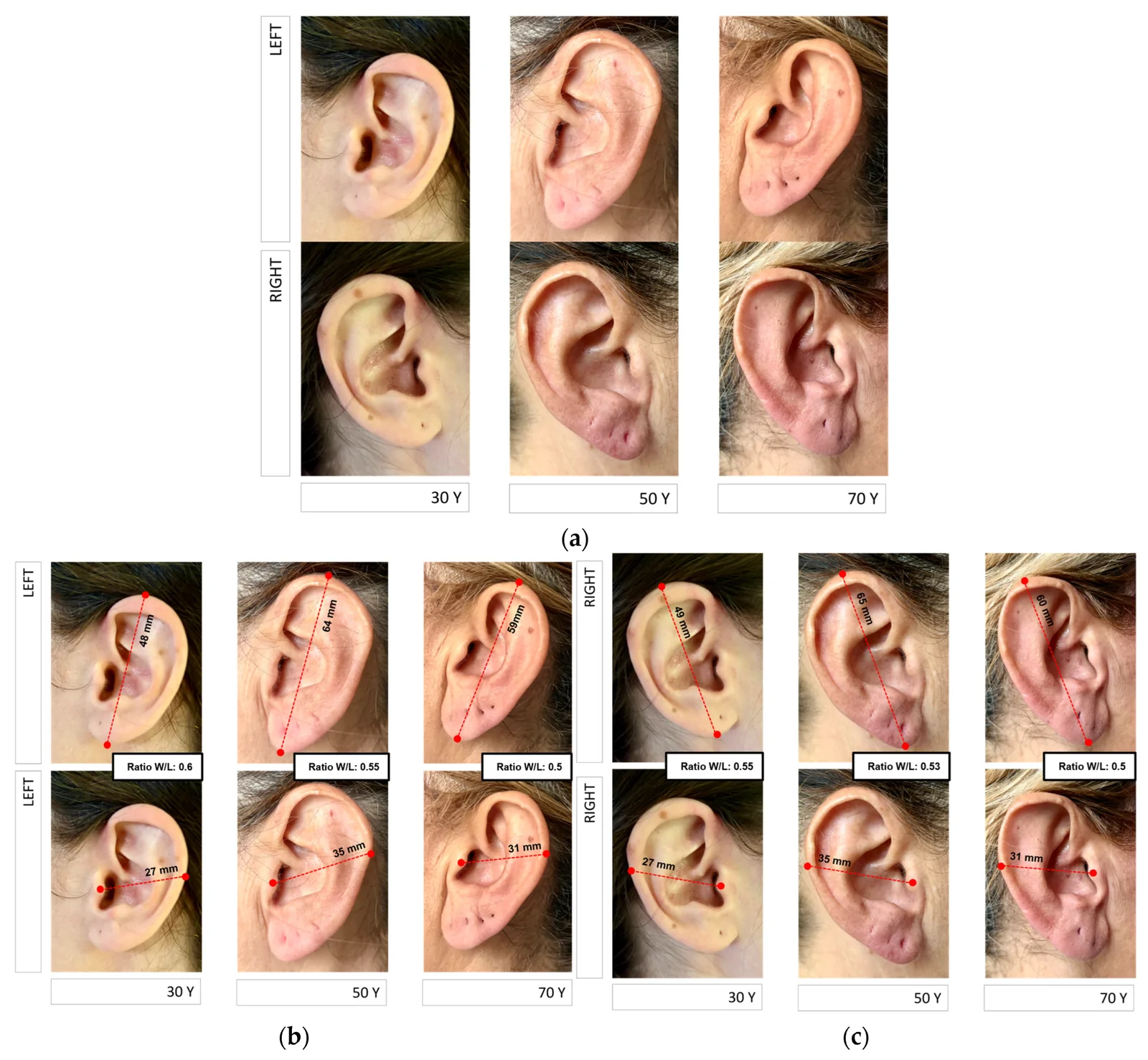
Grades AEAC 1–3 of the aesthetic ear aging classification. (**a**) Both the dimensions and shapes of the auricles change. The auricle elongates. The helix and the earlobes change their shape. In particular, the earlobe elongates. There is also a change in the inclination and protrusion of the entire auricle; (**b**) measures of the length and width of the left auricle at different ages. The ratio between the length and the width decreases over time; (**c**) measures of the length and width of the right auricle at different ages. Even on this side, the width-to-length ratio decreases over time. It is observable that in the same person, the dimensions and the ratio between the dimensions of the two auricles are, generally, different, according to the literature [19,55,56].

According to this classification, grade AEAC 0 corresponds to a non-aged auricle. In AEAC 1, an initial change, correlated with an initial cartilage alteration, begins, but it is evident only with facial movements. In grades AEAC 2 and AEAC 3, deep morphological variations are evident. An example of the grades 1–3 is shown in Figure 2.

Finally, the aging process of the helix may be completely different from the rest of the auricle, according to its initial shape. Nonetheless, in general, the helix undergoes relaxation, and a well-arched structure tends to assume an abnormal shape.

### 2.2. Ear Muscles and Adjacent Muscles Involved in Ear Aging and Muscles to Avoid During a BoNT Treatment

In the ears, the muscles are small. Nonetheless, they play a crucial role in the aging process.

**Figure 3:**
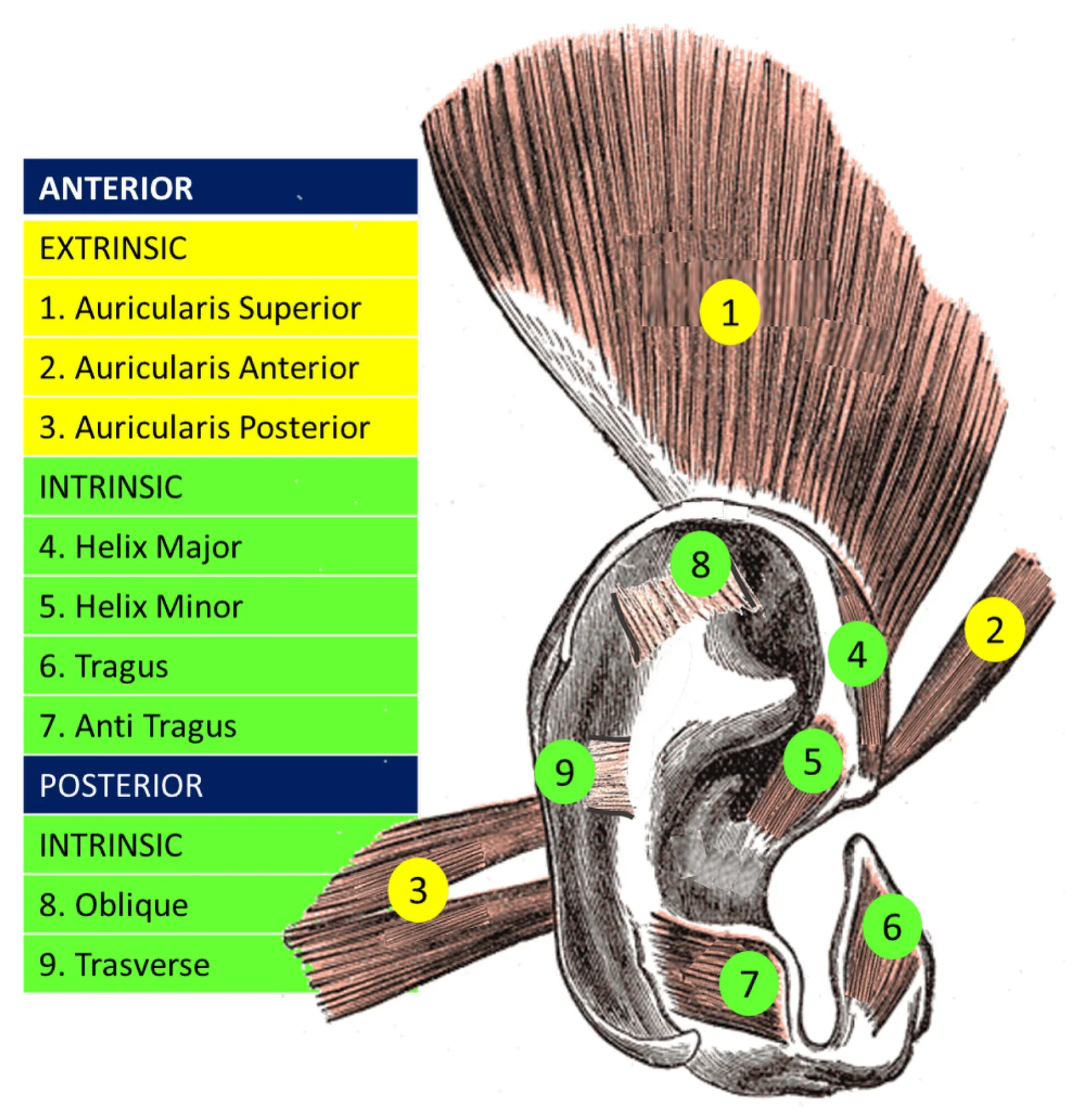
Muscles of the ear involved in the aging process. The extrinsic muscles are responsible for the inclination and protrusion of the entire auricle. The intrinsic muscles are involved in the shape of the auricle. Adapted from Anatomy of the Human Body (Plate 906—The muscles of the auricula), by H. Gray [57]. In the public domain.

The muscles of the ear include extrinsic and intrinsic muscles (Figure 3). The extrinsic muscles are the anterior, superior, and posterior auricular muscles. In front of the auricle, the anterior muscle pulls the ear forward. The superior muscle, above the auricle, slightly lifts the ear. The posterior muscle, behind the auricle, pulls the ear back. This means that the three extrinsic muscles contribute to the auricle’s positioning. The intrinsic muscles are the tragus and antitragus muscles, the major and minor muscles of the helix, and the transverse and oblique muscles of the ear. The tragus and antitragus muscles help define the contours of the ear. The major muscle of the helix is located on the anterior margin of the helix. The minor muscle of the helix is located on the outer part of the auricle and covers the crus, the cartilaginous part of the helix. Both muscles contribute to the shape of the auricle. The transverse and oblique muscles of the ear are located behind the auricle and help shape it. The effect of BoNT could potentially be exploited on all these muscles to reshape the auricle.

A particular note must be made regarding the helix, which is an arched cartilaginous structure, but whose tension is mainly influenced by the three auricular muscles. The superior auricular muscle is involved in the superior tension of the auricle. The posterior auricular muscle stabilizes the auricle and influences its posterior projection. The anterior auricular muscle exerts anterior traction, which lengthens the helix.

Other muscles, adjacent to the auricle, should be avoided during BoNT treatment. These include the occipital belly of the occipitofrontalis muscle, the temporalis muscle, and the mimic muscles near the ear. Although inadvertent injection of these muscles is unlikely due to their anatomical location, and the doses typically used around the ear are generally too small to produce clinically significant effects, awareness of these structures remains important. The occipitofrontalis muscle should be avoided to prevent compromising facial expression. The temporalis muscle should not be injected to avoid compromising chewing and preventing functional impairment. Mimic muscles, such as orbicularis oculi, should be avoided to prevent side effects, including ptosis. Finally, it is preferable to avoid injecting the masseter muscle to prevent chewing difficulties.

### 2.3. Results of Applying BoNT for Anti-Aging in the Auricles

The 50 subjects treated with Botulinum Toxin type A (BoNT-A) in this study were all female, aged 50 to 65 years. They presented with facial and auricular wrinkles associated with facial aging and sought a face-and-ear rejuvenation program at the center. The results of the BoNT-A selective neuromodulation treatment, applied according to the Russo Ear Lift Technique, of the auricles of these subjects with grades AEAC 1–2 may be described in terms of the effects on the different muscles.

Treatment of the superior, posterior, and anterior auricular muscles reduced muscle traction and caused retraction of the auricle. BoNT-A in the superior and posterior auricular muscles determined a reposition of the ear. Furthermore, in the superior auricular muscle, BoNT-A reduced wrinkles over the auricle. Finally, in the anterior auricular muscle, BoNT-A permitted the correction of the inclination of the auricle.

The treatment of superior, posterior, and anterior auricular muscles also reduced the tension of the helix cartilage and caused a slight folding of its superior and lateral portions. This resulted in an inward folding of the helix. Consequently, there was an overall effect on the entire auricle, which appeared more adherent and compact, reducing its prominence.

Toxin injections into the tragus and antitragus muscles were performed to harmonize the auricle profile with the zygomatic region.

Finally, treatment of the major and minor muscles of the helix reshaped the auricle and corrected wrinkles.

The overall effects of the treatment in the 50 subjects are summarized in Table 2.

**Figure 4:**
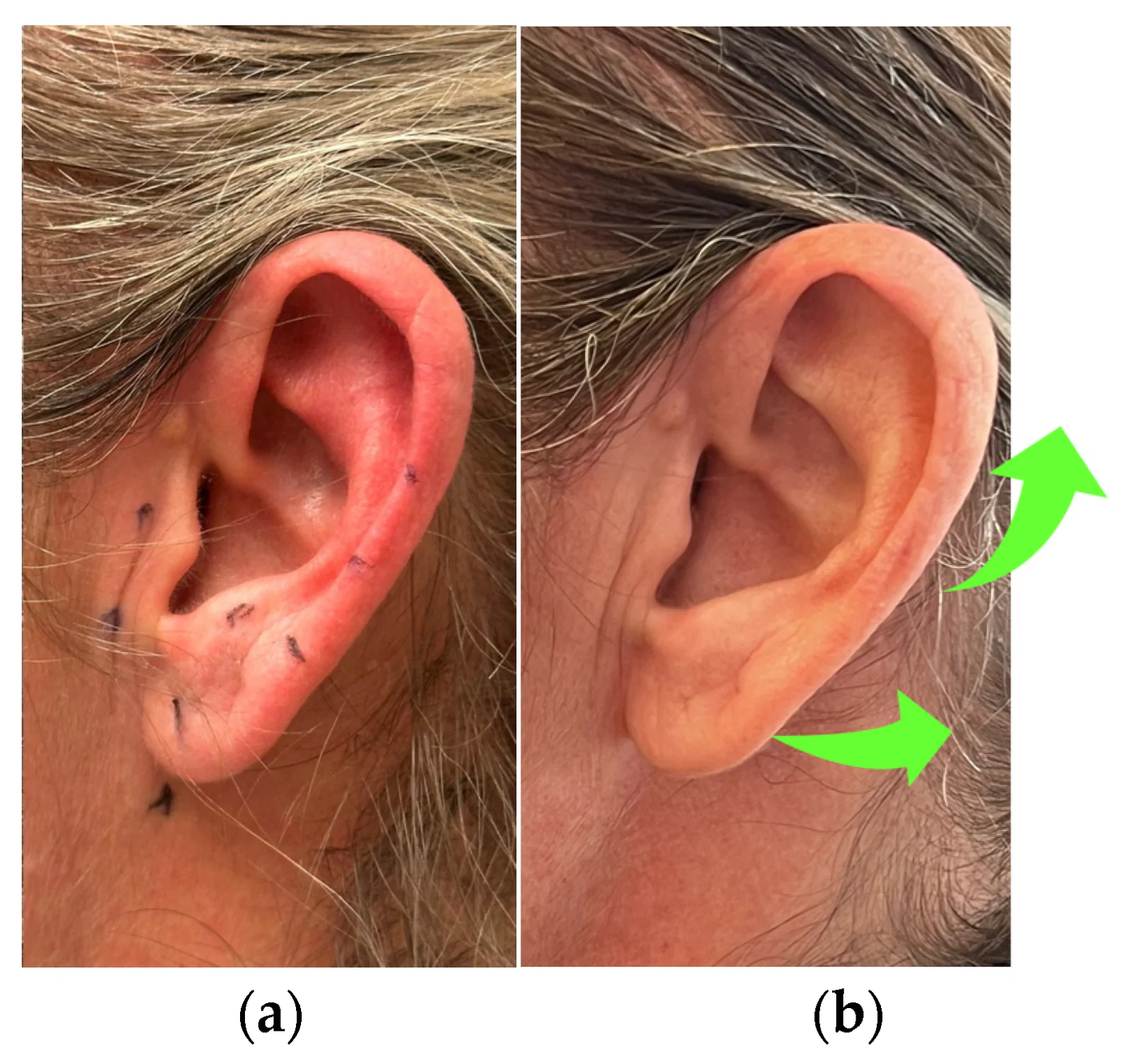

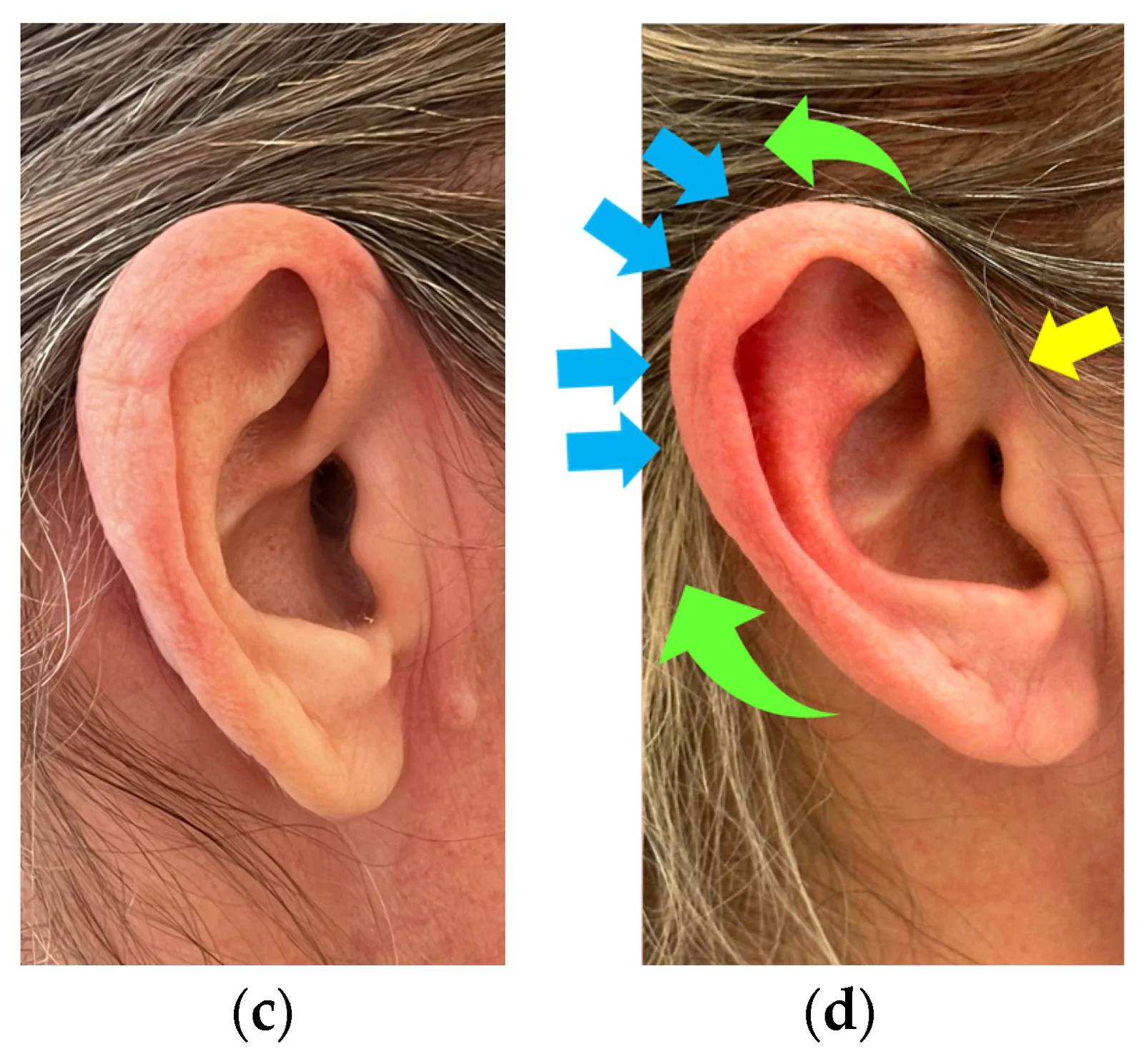
Effect of the BoNT-A selective neuromodulation treatment in two different auricles. (**a**) Left auricle before the treatment. The auricle is slightly protruded forward, and the superior part of the helix leans down; (**b**) left auricle after the treatment. The auricle is pulled back and slightly upwards (the green arrows highlight the correction); (**c**) right auricle before the treatment. The auricle is protruded forward and downwards, the helix shape is altered, and the concha results in enlargement; (**d**) right auricle after the treatment. The auricle is pulled back and slightly upwards, as the left auricle (green arrows). The helix shape is restored (blue arrows), and the porion is enlarged (yellow arrow), narrowing the concha.

**Figure 5:**
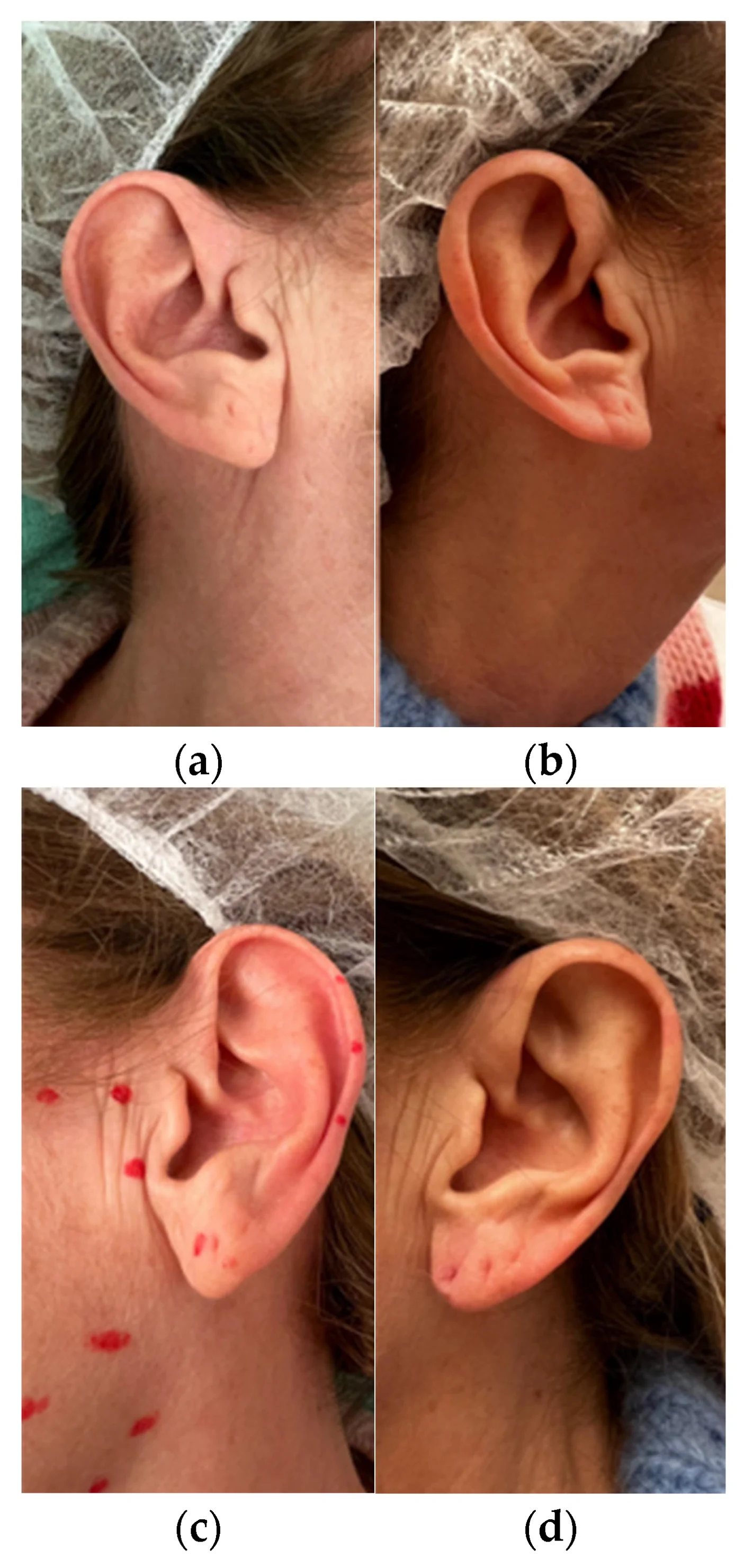
Effect of the BoNT-A selective neuromodulation treatment in two different helices. (**a**) Left auricle before the treatment. The helix is thinned, leaving the anti-helix completely uncovered; (**b**) left auricle after the treatment. The helix structure is restored and seems plump; (**c**) right auricle before the treatment. The helix is thinned, and the entire auricle protrudes forward; (**d**) Right auricle after the treatment. The helix shape is restored, and the auricle is pulled back.

Examples of the effect of the BoNT-A treatment on the auricle are presented in Figure 4. Details of the helix correction are shown in Figure 5.

Concluding, it may be stated that the rejuvenation effect of the selective neuromodulation with BoNT-A, applied according to the Russo Ear Lift Technique, in the extrinsic muscles is of auricle repositioning, while the action on the intrinsic muscles is the reshaping of the auricle. Nonetheless, the action of extrinsic muscles redistributes the mechanical load on the cartilage, resulting in minimal passive reshaping.

The overall effect was noticeable for 4 months in all patients, except 5 subjects, who showed it for 8 months.

The two types of toxins used produced the same results.

### 2.4. Side Effects

In the patients’ charts reviewed for this study, mild swelling and transient bruising at the injection site immediately after treatment were observed in 2 of 50 subjects (3 ears) (1.5% of treated ears), and resolved within 2–3 days. No other side effects were reported in the analyzed cohort. Moreover, no transitory asymmetries between the two auricles correlated with a non-perfect dosage of the product were observed.

### 2.5. Levels of Satisfaction

Although no satisfaction level assessment test was used, all patients required the treatment to be repeated when the aesthetic effects reduced or vanished. All 50 subjects reported that they used to keep their hair long as a hairstyle choice and to hide their ears, which caused them some embarrassment. After the treatment, all participants reported a significant improvement in their self-esteem and began wearing ponytails. This evidence leads to the conclusion that the toxin’s effect on the auricle was deemed fully satisfactory by all treated subjects.

### 2.6. Efficacy Evaluation

**Figure 6:**
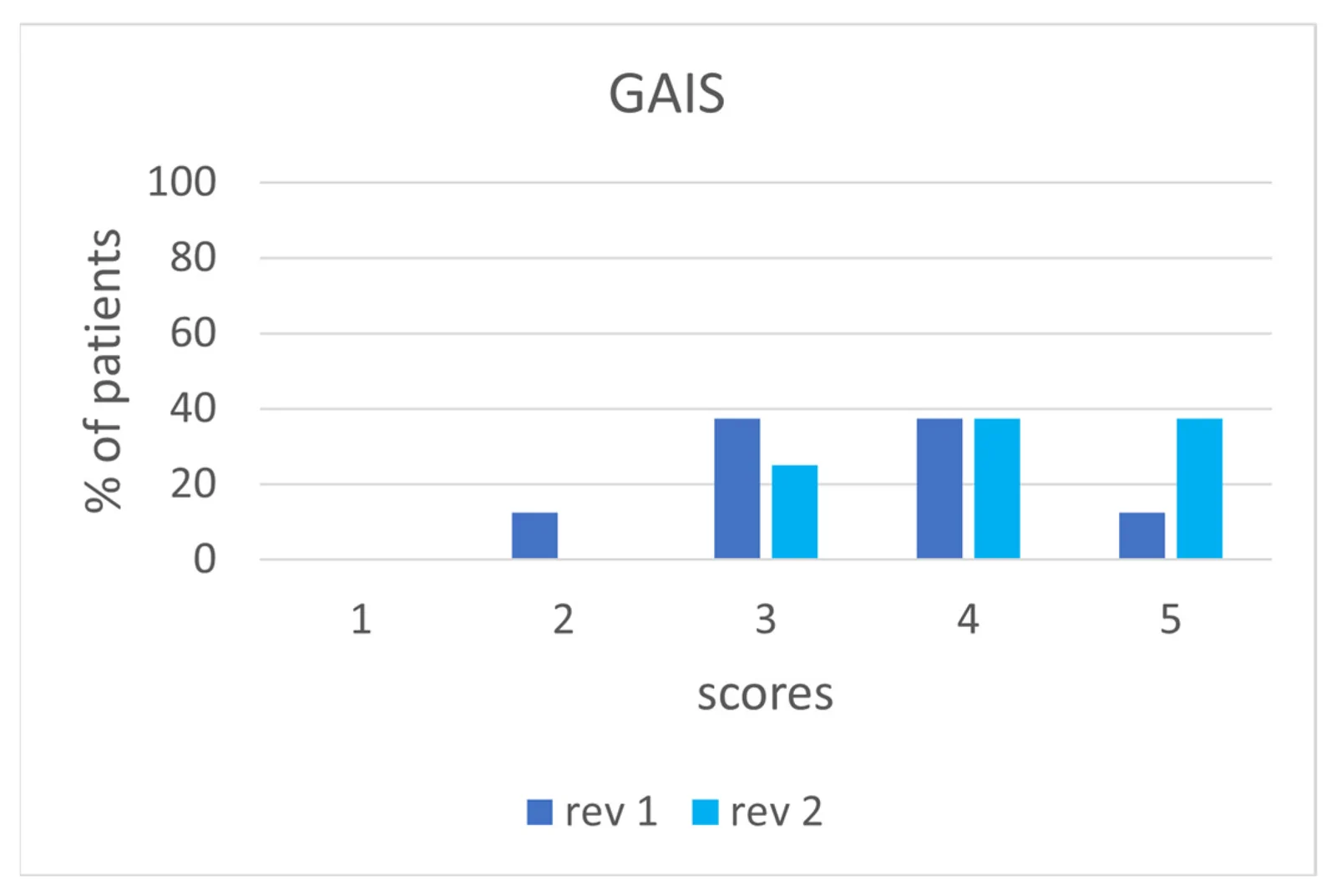
GAIS scores of the two reviewers of the treatment results. rev = reviewer.

The descriptive and comparative analyses of the Global Aesthetic Improvement Scale (GAIS) assessments demonstrated an overall positive clinical perception of the treatment from both blinded reviewers. The first reviewer reported GAIS scores ranging from 2 to 5, with a mean score of 3.50 ± 0.93 (median: 3.5), while the second reviewer reported scores ranging from 3 to 5, with a mean score of 4.12 ± 0.83 (median: 4.0) (Figure 6). The mean overall GAIS score, calculated from both reviewers, was 3.81 ± 0.66, indicating a generally favorable aesthetic improvement following treatment.

Comparison of reviewer scores using the Wilcoxon signed-rank test revealed no statistically significant difference in ratings between the two evaluators (W = 6.0, *p* = 0.160). Although the second reviewer tended to assign slightly higher scores than the first, the difference did not reach statistical significance.

However, inter-rater reliability analysis demonstrated limited agreement between the two evaluators. The exact absolute agreement between the two was 12.5%. The linear weighted Cohen’s kappa coefficient was κ = 0.070, indicating poor inter-rater agreement, that is, limited concordance in the magnitude of aesthetic improvement assigned to individual patients. Similarly, the single-rater absolute agreement coefficient, the Intraclass Correlation Coefficient (2,1), was ICC(2,1) = 0.081, and the consistency coefficient, the Intraclass Correlation Coefficient (3,1), was ICC(3,1) = 0.092. Both values consistently indicated a poor inter-rater reliability, reflecting noticeable individual variations in subjective cosmetic grading across the cohort. Nonetheless, both reviewers uniformly judged the treatment as producing aesthetic improvement across the study population.

## 3. Discussion

Treatment of the auricles with BoNT-A for aesthetic corrections might not be considered essential. In fact, even if the auricles play a fundamental role in the hearing process, as they capture the acoustic wave and provide a first amplification of 30–100 times the sounds at 3000 Hz [58,59], and they are essential for localizing the acoustic source [59,60], auricle aging seems not to influence hearing perception. Surgeries for anomaly correction are recommended only in cases of severe malformations, mainly congenital, that are associated with hearing disorders [61]. Nonetheless, given the psychological impact of auricular abnormalities on people [4,5] and the benefits of their correction [52,53], treatment with BoNT-A for aesthetic correction is not only useful but necessary.

According to Colombo et al. [62], the auricle shape is a defining characteristic of the face and, by citing Boesoirie et al. [56] and Buyuklu et al. [18], is as unique as human fingerprints and determines the face’s appearance. Consequently, it is commonly believed that ear rejuvenation must be performed as an integral part of the rhytidectomy. It must be added that rhytidectomy alone may cause ear deformities, such as the so-called “evil or pixie earlobe” [62]. This type of side effect implies a sequential corrective ear surgery. The procedure is often called an “ear lift” and primarily refers to the earlobe. The skin excess is removed, the lobe is re-shaped, and the scar is hidden in natural creases [63].

The aging process of the auricles is mainly associated with tissue changes [16,20,24,26,64,65] and gravitational forces [21,62,66]. Anti-aging treatments have focused solely on the earlobe, although the debate remains open as to whether the elongation of the ear with age is due solely to the lobe alone or to the entire auricle [26,67,68]. The non-surgical treatments developed to date include volume restoration, sagging minimization, improvements in texture and tone [45,66], and corrections for wrinkling and hyperpigmentation [45,62] at the earlobe level. HA fillers are generally used for volume restoration and to minimize sagging [69,70,71]. For photoaging, treatments such as tretinoin application, topical application of HA, chemical peels, carbon dioxide (CO2) laser, and light-based therapies are preferred [72,73]. Although all these procedures demonstrate that ear aging is a relevant problem and that an ear’s aging pattern is already known, no aesthetic classification has been proposed. In this paper, the authors defined an AEAC to facilitate treatment selection.

The role of the muscles in the ear’s aging process seems to have been overlooked or underestimated, unlike that of the face [74,75,76]. In the present study, the authors have highlighted how the auricle’s muscles function and their contribution to ear ageing. Extrinsic muscles are involved in the ear’s position, contributing to the inclination and protrusion of the auricle. Intrinsic muscles are involved in the maintenance of the auricle’s shape. Consequently, it can be stated that the aging process is associated with structural changes, sagging, and alterations in muscle contraction.

The other main aim of this study was to demonstrate how the use of the BoNT-A on the auricle’s muscles permits the modulation of their strength, reducing their depressor activity and restoring their balance effect. Thus, in this particular treatment, the toxin was not used to immobilize the muscles involved in the aging process, as occurs, for instance, with the forehead muscles [77]. The calibrated dosage of the toxin in the extrinsic muscles, in particular, was fundamental for the balancing of the tractor force of these three muscles. For instance, the protrusion of the auricle depends on the balancing of the anterior and posterior auricular muscles, while the action of the superior auricular muscle seems mainly of auricular stabilization. A protrusion of the auricle was referred to as a dominance of the anterior muscle over the posterior. However, the only treatment of the anterior muscle would not guarantee a proper correction of the ear positioning, because the posterior muscle seems to have a slight rotational effect on the auricle. For this reason, all the auricular muscles should be treated to restore the equilibrium of the different traction forces.

The clinical efficacy of the treatment is correlated with the just-mentioned equilibrium of muscles’ traction forces. The aging process of the auricle involves changes in its position and shape, as detailed in Table 1 and illustrated in Figure 4a,c and Figure 5a,c. The treatment seems to restore the broken equilibrium, and the overall effect is a correction of the auricle axes, the auriculocephalic angle, the distance between the helix and the mastoid, the ear protrusion, and the auricle shape (Figure 4b,d and Figure 5b,d). Depending on the ageing grade, a total or partial correction is achieved, thereby clarifying the clinical indication. It is clear that, according to the introduced classification, grade AEAC 0 should not be treated with BoNT-A, or, if preventive treatment is chosen, a minimal dose must be used, because it induces ephemeral modifications. Conversely, ears classified into one of the other three grades may benefit the most from treatment. For grade AEAC 1, dosage can be reduced, and the treatment results in an ear lift. The grade AEAC 2 seems to be the ideal phase to treat. Anti-aging effects are evident. Nonetheless, to improve the skin texture, a booster should also be added, that is, a hyaluronic acid filler with a low storage modulus (G’) and a high tan delta (the loss factor in rheology). This type of product is ideal for superficial areas because it is elastic, viscous, and fluid-like. The AEAC 3 degree is the most complex to treat, and benefits may be limited, depending on the degree of sagging and earlobe alterations. The elongation of the auricle that correlates with aging may not be prevented or modified by toxin use. In this case, only a filler or biostimulator might be used to volumize the earlobe, and a hyaluronic acid product with a higher G’ has to be preferred. Overall, the treatment cannot solve the sagging problem. Its main effects are the realignment of the auricle, the reduction of the auriculocephalic angle, and the reshaping of the auricle. In patients with advanced skin laxity, the authors believe the treatment may be ineffective or have a limited effect.

The expected side effects in this study may include those commonly observed with BoNT-A treatment [78,79,80]. Therefore, mild swelling or bruising at the injection site immediately after treatment was considered possible. Side effects reported in the literature for other ear treatments include those associated with earlobe rejuvenation. Generally, treatments in this area are considered safe [48]. The earlobe presents a rich capillary plexus, which ensures adequate vascularity, even in the case of a vascular occlusion with a filler. However, bruising, swelling, and local infections may occur [49,63,70]. Di Gregorio and D’Arpa reported transient erythema [81]. The recovery time for edematous swelling was 3–4 days in Arora and Arora [49], whereas Qian et al. [70] reported that the bruising required 7 days to resolve. Arora and Arora also warned about the possible formation of nodules after a filler. For small nodules, a natural resolution within 4 weeks was reported. Nonetheless, the topical use of Non-Steroidal Anti-Inflammatory Drugs (NSAIDs) and Heparin may help. In many cases, the nodules are hematomas. So, a light daily massage can help shorten the resorption time. In extreme cases, hyaluronidase dissolution might be necessary. Recently, autologous fat transfer has been suggested, but only for earlobe treatment [63,82,83]. For this procedure, no side effects were reported at the earlobe level. Nevertheless, this technique must be performed in a surgical clinic, and side effects correlated with fat harvesting must be taken into consideration.

In the present study, the incidence of a transient effect was 1.5%, lower than that reported in the literature for facial BoNT treatments [79,84]. In particular, Santorelli et al. [79] reported bruising at the injection site, temporary headache, eyebrow ptosis, and dry eyes. The incidence of bruising was 1.5%, as in the present study. It is clear that the other types of side effects described by these authors might not be experienced in the auricle treatment. The low incidence of side effects in the present study may be due to the injection sites. In fact, the muscles treated are vestigial, that is, muscles that during human evolution have lost their functionality. While animals exhibit significant and extensive movement, and the two auricles can move independently [85,86], human auricle muscles do not allow movement of the auricle or allow only minimal movement [87]. Their main effect is to maintain auricle position, probably to facilitate hearing. In the area, no other muscles were present. So, the risks were extremely low, particularly with respect to facial movement. When injecting into the anterior auricular muscle, special care must be taken to avoid diffusion into the masseter muscle. But the dosage used for ear treatment is extremely lower than that generally used for masseter treatment [88]. Therefore, an accidental diffusion of the BoNT-A product should not cause a real effect on the masseter and on the chewing function. Assessing the strength of these muscles is extremely difficult, as it is not possible to perform the static and dynamic evaluations typically used when BoNT is injected. Moreover, even an electrophysiological evaluation is extremely complex and may have limited results.

Finally, even if it is not a true side effect, it is important to consider that a temporary asymmetry may be observed after the first 7–14 days, and this may cause discomfort in the treated subject. This is a common transitory phenomenon that resolves on its own. In fact, if the dose is sufficient and symmetry was achieved during the treatment, the presence of asymmetries should be considered exceptional after 14 days. In these exceptional situations, a finishing touch would be required. In the present study, no transitory asymmetries were observed. This is not a banal result because the two auricles are normally asymmetric and may exhibit different degrees of ageing [89]. So, the dosage calibration must be carefully defined and may differ between the two auricles.

Overall, the treatment offers the indisputable advantages of a BoNT treatment: it does not require anesthesia, has no recovery time, is outpatient, is quick (15–30 min), and, if the patient is unsatisfied, is not permanent, with corrections and modifications possible, or the procedure can be aborted.

Regarding satisfaction levels among treated women, even without a proper scale to assess them, the request for a repeat treatment at the end of the toxin’s effects is extremely significant. The psychological benefits of ear procedures for patients have already been documented [54], and this data appears to be confirmed by treatment with the toxin.

Finally, the treatment’s efficiency evaluation confirmed the aesthetic improvement of the ear. Despite the novelty of a rejuvenation treatment for the entire auricle rather than just the earlobe, the two reviewers noted improvement. The differences in grading between the GAIS scores of the two reviewers may be correlated with both the novelty of the treatment and the lack of a clear, well-defined aesthetic and aging classification of the auricle. The positive overall evaluations seem to confirm the methodology’s efficiency, but the presence of scores 2 and 3 suggests the need for an accurate and appropriate selection of patients to treat.

The limitations of this study for the treatment evaluation include the small number of cases reported and the fact that they were treated at a single clinic by a single surgeon. In reality, from a different perspective, this last aspect might be considered an advantage of the study, as it eliminates bias arising from practitioners’ diverse experiences. Another limitation of the present study is the narrow age range of the subjects treated, which ranged from 50 to 65 years. It would also be interesting to understand why only women patients in this age range requested the ear rejuvenation treatment with BoNT-A at the center. This may depend on many factors. Although the literature reports that cosmetic ear surgery is particularly appreciated by men [52], at the center where the treatment was performed, requests came only from women. It would be interesting to understand if men prefer more definitive treatments, such as surgery, than transitory treatments, such as the BoNT one. Finally, even if the results indicated a high level of patient satisfaction, using a standardized scale would enable an objective evaluation of this extremely important aspect.

Undoubtedly, further studies are required to outline age-related guidelines. Moreover, it may be helpful to measure the patient’s satisfaction using a proper scale. Last but not least, a stricter definition of the characteristics of the patients to be treated might be useful to ensure a GAIS score of at least 4 when evaluating aesthetic effects.

## 4. Conclusions

This study presented a selective neuromodulation BoNT-A treatment, the Russo Ear Lift Technique, which was not previously described in the literature. It is used as a complementary procedure to the common facial treatment. To date, no major side effects or complications have been reported. The treated subject’s satisfaction levels were high, as evidenced by requests to repeat the treatment. To apply it, attention to possible anatomical variations and proper dose management are essential for preventing complications and optimizing treatment efficacy. Based on the results and the classification introduced, the procedure appears ideal for managing AEAC grades 1 and 2. In contrast, for grade 0, the authors suggest preventive measures, such as sunscreen (for photoaging) and hydrant products (for natural aging). In grade 4, associating the procedure with other aesthetic procedures, such as fillers (particularly for earlobe volumization) and photoaging treatments, when hyperpigmented macules are present, might improve aesthetic outcomes and yield optimal results. Nevertheless, as ear aging is highly individual and depends on many intrinsic and extrinsic factors (nutrition, sun exposure, smoking, drug use…), even in grades 1 and 2, combining it with other aesthetic treatments may be necessary to achieve good results.

It is possible to conclude that the treatment has been shown to be clinically effective in correcting ear aging, has demonstrated a favorable safety profile, is associated with high levels of satisfaction and improved quality of life among those treated, and has been positively evaluated by blinded experts. To better validate the treatment, further studies with instrumental measurements, such as 3D reconstruction of the auricle’s initial and final positions, are possible. The proposed classification can help with the choice of anti-aging auricle treatment. As already highlighted, different levels may benefit from different solutions, and the optimal solution for one level may not be the same for another. Further studies are needed to optimize treatments for each grade.

## 5. Materials and Methods

This retrospective study was conducted at a single center and included patients treated with BoNT type A in 2024; subjects were monitored for the duration of the effects. The data were obtained from the patients’ medical records. The primary outcome of this study was the evaluation of the aesthetic implications, efficacy, and safety of selective neuromodulation with BoNT-A application to the auricles, named Russo Ear Lift Technique. This result was achieved by passing through the secondary outcomes of aesthetic classification of auricle aging and anatomical evaluation of the muscles involved in this process.

### 5.1. Aesthetic Classification of Auricular Aging Study

To define the Aesthetic Classification of Auricular Aging (AEAC), a cross-sectional observational study was conducted. Data from 40 subjects (20 women and 20 men), aged 30–68, were selected from medical records. The records had to contain pictures of both auricles taken with the heads aligned in the Frankfurt horizontal plane. The records were selected sequentially over time (by date of admission to the clinic, starting at the beginning of 2024) to ensure equal representation of four age groups (30–39 years, 40–49 years, 50–59 years, and 60–69 years), divided by sex. Therefore, a group of five subjects was defined for each age-sex combination. For each patient, two measures were evaluated for each ear:

- the length, that is, the distance between the supra-aurale (highest) point and the subaurale (lowest) point of the auricle;
- the width, that is, the distance between the tragus and the postaurale (most posterior or backwards-most) point of the auricle.

To take the measures, a millimeter ruler was used.

Then, the auricular index, that is, the ratio between the width and the length of each auricle, was calculated.

The data were collected and analyzed using descriptive statistics, and Microsoft Excel (version 365, Microsoft Corporation, Redmond, WA, USA) was utilized. No inferential statistical analyses were performed. The mean and standard deviation of the length and width measures of each group were calculated.

The auricle aging pattern of the entire cohort resulted in a pattern similar to that reported in the literature: the length of the auricles increased [18,19,55,67,68,90,91,92,93], while the width remained almost stable over time [55], and the auricular index decreased over time [90,92]. The observed similarities enabled the definition of an aesthetic aging model within the limited cohort of the present study, as these people were representative of the entire population; therefore, the model could be generalized to the entire population.

It is essential to note that, for some individuals, the aging process may begin earlier than for others, and that there are gender-related differences. Consequently, an aesthetic classification correlated with the age fascia of the 4 groups was not possible; however, four degrees of aesthetic aging were defined based on the severity and the structures involved.

Various aspects were considered to define the four degrees: the quality of the skin, the position of the auricles, their shape, and the shape of the earlobe.

Considering that young skin is tight, well-adherent to the underlying tissues, and wrinkle-free, its aging was evaluated by assessing thinning through the appearance of capillaries and the formation of wrinkles. In particular, capillaries become evident where the auricles are thinner, that is, at the level of the helix and antihelix. Here, the perforating branches of the superior auricular and posterior auricular arteries are present [94].

Ear position was evaluated through the measures of the auricle’s inclination, protrusion, and the distance between the helix rim and the mastoid. The inclination was calculated by measuring the angle formed between the medial longitudinal axis of the auricle and the vertical [92]. The auricle is normally rotated posterolaterally by 15–30° [89,95,96]. The protrusion was evaluated using the auriculocephalic angle, which is calculated by measuring the angle between the upper posterior part of the auricle and the mastoid [92]. Normal values are <25° in men and <21° in women [97]. Finally, the distance between the helix rim and the mastoid was calculated. In the literature, the measure is described from the mastoid to three different points: the superior and middle points of the auricle, and the earlobe. In these three points, the reference values are 10–12 mm, 16–18 mm, and 20–22 mm, respectively [95,96]. With aging, the auricle tends to rotate forward, increasing the axis and the protrusion. This last implies a decrease in the auriculocephalic angle [89]. A 5° difference was used to classify different degrees of ear aging, based on both the auriculocephalic angle and the angle between the medial longitudinal axis of the auricle and the vertical. Considering the deformations that aging can cause at the rim of the helix, to evaluate the distance between the auricle and the mastoid, the midpoint of the auricle was taken into account, and a 2 mm increase was set as the limit for stadiation.

Ear shape is correlated to concha dimensions, helix shape, and cartilage alterations. The conchal bowl is normally hemispherical and extends to a depth ≤ 1.5 cm. With aging, as the auricle protrudes, the concha rotates anteriorly and deepens [89,95,96]. Thus, this limit value was considered to define aging. The helix shape is correlated with the cartilage alterations. Normally, the helix begins in the concha and ends at the level of the intertragic notch, forming an incomplete, curvilinear tube, 5–8 mm wide [13]. Aberrations of the helix are very common [92]. With aging, an increase in cartilage stiffness (Young’s modulus) was observed, accompanied by progressive deformation or lengthening of the auricular structure [20]. Consequently, both flat and wide helices have been described, and the helix may open and become thinner or drop, widening [89,92]. The type of change was correlated with the patients’ age.

The shape of the earlobe, and especially its elongation, was assessed using Mowlavi’s scale as a reference. Earlobe length pleasantness was measured from most desirable to least pleasant as 5 mm > 10 mm > 0 mm > 15 mm > 20 mm. The 10 mm measurement is the surgical limit [43,44]. To define the variation in earlobe aging, given its high length variability, a 5 mm difference was hypothesized to indicate the degree of aging.

Finally, an asymmetrical position between the two auricles is commonly observed and considered normal within a limit of 3 mm in both horizontal and vertical positions [89,97]. With aging and cartilage modifications, the asymmetry may worsen. A value greater than 3 mm has been taken as a reference value to highlight a worsening auricle asymmetry.

### 5.2. BoNT-A Treatment Study

To assess the aesthetic implications and efficacy of the BoNT-A treatment, a consecutive retrospective cohort study was conducted. The study was neither controlled nor randomized, as only outcomes were observed. The sampling was non-probability, with all consecutive patients included to reduce the risk of selection bias. The charts of the first 50 subjects treated with BoNT-A from the beginning of 2024, with a 1-year follow-up, were considered. To evaluate the safety of the treatment, the incidence and type of adverse effects were collected and compared with those reported in the literature on facial treatment with BoNT-A.

Given the time to action of BoNT-A (results visible within 7–14 days after injection), monitoring of the aesthetic effect was scheduled at 7, 14, and 30 days to allow minor corrections (new injections) if necessary. Moreover, monitoring was performed at 90 and 120 days to assess the effect of reduction and to administer any necessary additional BoNT-A injections upon patients’ request. If the effects lasted longer, further monitoring was scheduled.

Patients were required to report any side effects immediately, and adverse effects were collected retrospectively from clinical charts.

A visual evaluation of changes in auricular morphology and positioning was performed by comparing pre- and post-treatment photos of the subjects involved in the study. The effects on individual muscles were derived from comparisons of results with the different muscles’ roles and their involvement in the aging process.

Patient satisfaction was assessed indirectly by considering the number of patients who requested repeat treatment. Furthermore, any changes in their perception of their ears were recorded.

Aesthetic efficacy was evaluated through a blinded, retrospective, objective analysis of pre- and post-treatment photographs. Two independent, experienced clinicians, who were completely blinded to the specific treatment protocols administered, reviewed the images. The reviewers were not provided with any information regarding patient characteristics, clinical outcomes, or study objectives. They assigned a score reflecting the degree of aesthetic improvement observed after treatment, using the standardized rating scale Global Aesthetic Improvement Scale (GAIS). This scale uses a 5-point Likert ranking to evaluate change: 1 worse, 2 no change, 3 improved, 4 much improved, 5 very much improved.

### 5.3. Inclusion and Exclusion Criteria for BoNT-A Treatment

The subjects selected had to meet the following inclusion criteria:

- No previous BoNT treatments;
- No facial or ear problems or abnormalities. This is in line with Fijałkowska et al. [98], who stated that subjects with ear-piercing-related or congenital abnormalities should not be treated.

Subjects were excluded from the treatment according to the contraindications of the BoNT injections [99,100,101,102]:

- Allergy or sensitivity to constituents of the product;
- Dermatoses or infections in the area to treat (e.g., eczema and psoriasis);
- Dysmorphic disorder;
- Gross motor weakness or neuromuscular disorders in the area to treat (e.g., Bell palsy and myopathies);
- Keloidal scarring;
- Immunocompromised;
- Pregnancy and breastfeeding.

All subjects were adequately informed about the procedure and its implications, and signed an informed consent to undergo the treatment.

### 5.4. Injection Technique

Five key points may be considered essential for reducing muscle tension and improving the aesthetic prolife of the auricle: the earlobe, the retroauricular sulcus, the antitragus, the upper margin of the tragus, the margin of the helix, and the preauricular area.

The Earlobe: Targeted injections can relax the folds, improving the appearance of the earlobe if it is wrinkled or flattened. Injections should be performed in the center of the earlobe to improve sagging skin and give the earlobe a fuller appearance. The upper and lower margins of the earlobe should be treated if marked folds are present.

The Retroauricular Sulcus: This area should be treated to relax overactive muscles, which cause skin tightness and the formation of wrinkles and folds. Injections should be placed along the sulcus, where the skin is thinnest.

The Anti-Tragus and the Upper Margin of the Tragus: Mild wrinkles and folds may develop in these areas. A small amount of BoNT can be injected into the visible folds to flatten these areas.

The Margin of the Helix: To improve the skin tone around the helix, injections should be performed along the helix’s outer margin.

The Pre-Auricular Area: This area, which is not part of the ear, can be treated to complete the treatment. Indeed, when performing an aesthetic treatment, it is essential to ensure aesthetic continuity between the treated areas and the surrounding areas. Therefore, injections can be performed near the pretragal region of the ear, where horizontal wrinkles can develop.

Two types of BoNT-A were used: abobotulinumtoxinA (ABO) (Dysport, Ipsen Ltd., Slough, UK/Azzalure, Galderma SA, Lausanne, Switzerland; 125 U vials) and onabotulinumtoxinA (ONA) (Vistabel/Botox, Allergan, Inc., Irvine, CA, USA; 50 U, or 100 U vials). Before injection, the BoNT-A was diluted with saline at a ratio of 125 Speywood Units of ABO/0.63 mL of saline, and 100 Allergan Units of ONA/2.5 mL of saline or 50 Allergan Units of ONA/1.25 mL of saline [100,103,104,105]. 

**Figure 7:**
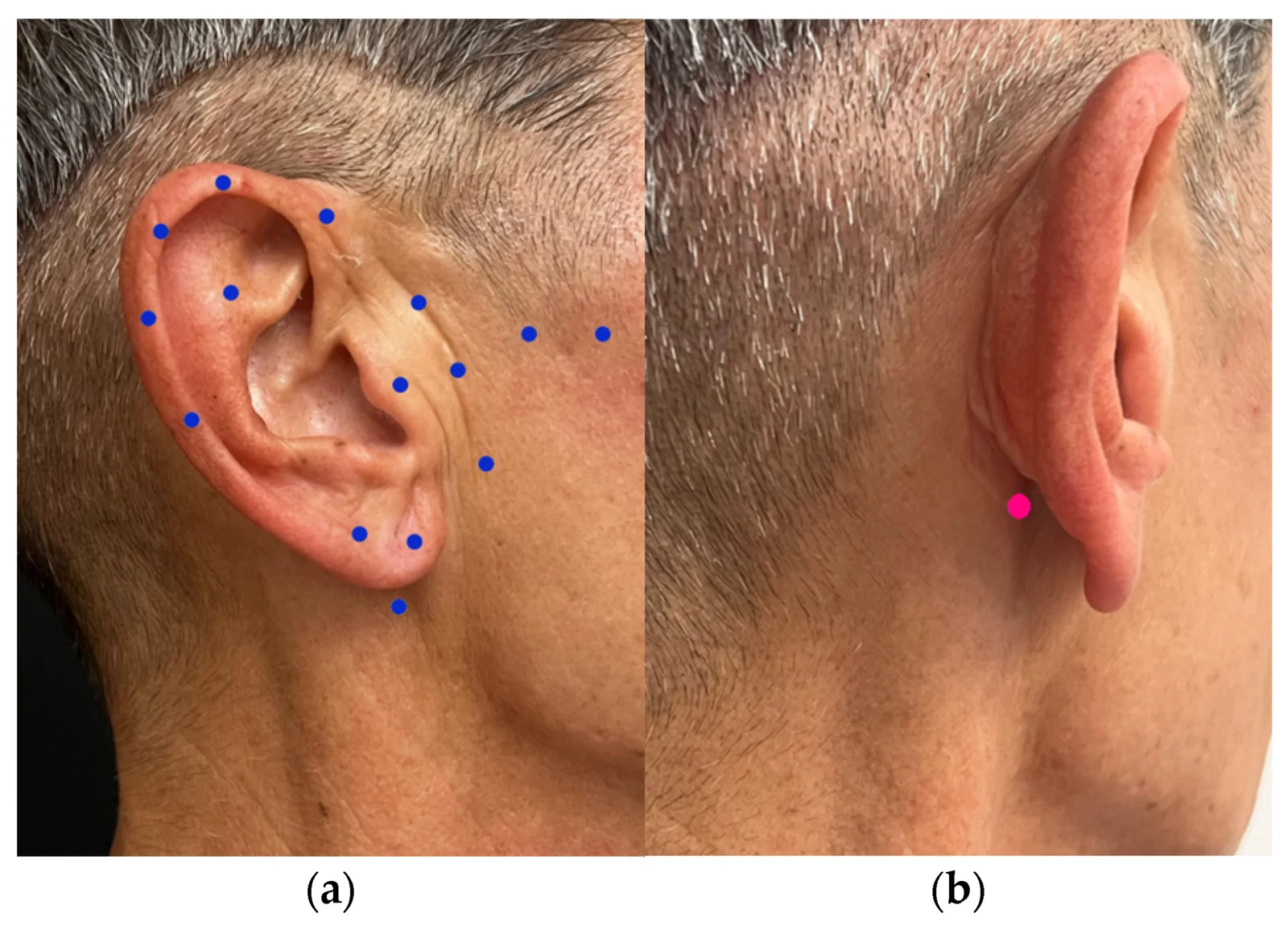
Inoculation points of the BoNT-A, according to the Russo Ear Lift Technique. (**a**) The anterior part of the auricle is treated in multiple points to correct both the auricle position and the alteration in its shape; (**b**) posteriorly, there is a single injection point on the auricularis posterior muscle, to correct the auricle’s protrusion.

Injections were administered intramuscularly or intradermally with a 30-gauge needle. The points of inoculation, their depths, dosages, effects, and possible side effects are schematized in Table 3. The inoculation points are summarized in Figure 7.

For correcting only the helix, an injection in the superficial area above the ear, 1 cm from the superior insertion line, was performed. This injection was superficial/subdermal to avoid deep diffusion. The dosage was 1–2 U ONA/3–5 U ABO per point, per side of the head.

If a shape refinement was required, injections were performed at the earlobe and sub-lobular areas. In both cases, injections were superficial/subdermal. In the earlobe, they were performed in 2 points, while in the sub-lobular area, they were performed in a single point. A dosage of 1–2 U ONA/3–5 U ABO per point was used in both sites.

A total dosage of 25–50 Speywood Units per ear was used for ABO, and 12–24 Allergan Units per ear was used for ONA. The dosage required in the ear area was generally minimal, as the muscles being treated are of small dimensions and thin. In any case, treatment had to be carefully tailored to the patient’s needs and the anatomical structure of their ears. Evaluating any asymmetries between the two auricles and adjusting the dosage based on the required correction was necessary. The dosages were defined according to the conservative micro-dosing principles [38,100].

After injections, a gentle compression was applied if needed. No pressure or massage was applied to the treated area, and treated subjects were instructed to avoid doing so for the first 24 h following treatment, as recommended by Santorelli et al. [79].

### 5.5. Statistical Analysis of Efficiency Evaluation

In the treatment efficiency evaluation, the GAIS scores from the two blinded reviewers were summarized using descriptive statistics and reported as mean ± standard deviation (SD), median, and range. A Wilcoxon signed-rank test was utilized to detect systematic shifts or significant differences between the two reviewers’ scoring distributions, that is, to identify any systematic tendency of one reviewer to assign higher or lower scores than the other. A two-sided *p*-value < 0.05 was considered statistically significant. The inter-rater agreement between the two reviewers was evaluated using Cohen’s weighted kappa (κ) with linear weighting coefficient, and the Two-Way Mixed Effects Intraclass Correlation Coefficient (ICC), considering the two reviewers as fixed raters, and patients as random effects. The κ coefficient was interpreted according to the Landis and Koch classification: <0.00, poor agreement; 0.00–0.20, slight agreement; 0.21–0.40, fair agreement; 0.41–0.60, moderate agreement; 0.61–0.80, substantial agreement; 0.81–1.00, almost perfect agreement [106]. ICC values were interpreted according to commonly accepted criteria: <0.50, poor reliability; 0.50–0.75, moderate reliability; 0.75–0.90, good reliability; and >0.90, excellent reliability.

All statistical analyses were performed using Python version 3.12 (Python Software Foundation, Wilmington, DE, USA) with the SciPy and scikit-learn statistical libraries.

## Figures and Tables

**Table 1:** Aesthetic ear aging classification (AEAC).

Grade	Main Signs	Cartilage Modifications	Earlobe Modifications
AEAC 0	Well-adherent and tight skin Stable axis Normal auriculocephalic angle	No changes (stable helix and antihelix)	No changes
AEAC 1	Skin still normotrophic Shallow concha Only with facial movements, dynamic alterations and changes in ear protrusion	Minimal reduction (1–2 mm descent of the upper helix and auricle with a more “open” appearance laterally)	Mild thinning and lengthening of the earlobe (ptosis ≤ 5 mm from basal length)
AEAC 2	Evident prolapse of the auricle (axis modification: increase of inclination < 5° and reduction of the auriculocephalic angle < 5°) Increase in the distance between the helix and the mastoid (≤2 mm) Appearance of thin preauricular wrinkles (preauricular skin laxity) Frequently, asymmetry between the two auricles (>3 mm in the horizontal and vertical position)	Loss of definition of the lateral profile of the ear (cartilage alteration; modification of the helix curvature and/or shape)	Thinned and elongated earlobe (ptosis of 5–10 mm compared to basal length)
AEAC 3	Marked auricular ptosis (axis modification: increase of inclination ≥ 5° and reduction of the auriculocephalic angle ≥ 5°) Increase in the distance between the helix and the mastoid (>2 mm) Stable ear protrusion alterations (not only dynamic) Capillaries visible at the level of the helix and superior crus (thinning of the skin) Preauricular wrinkles Deep concha bowl	Structural loss with alterations in the shape of the ear (cartilage elongation and/or alteration; collapse or opening of the helix > 2 mm)	Elongated, wrinkled, and deflated earlobe (lobar ptosis > 10 mm compared to basal length)

**Table 2:** Effects on the single muscles of the BoNT-A treatment.

Muscle	Area	Effect
Anterior Auricular	Upper preauricular region	Reduction of anterior ear traction
		Reduction of the protrusive effect
Superior Auricular	Upper auricular region	Auricle elevation
		Reduction of the area’s wrinkles
Posterior Auricular	Posterior auricular region	Improvement of posterior ear stabilization
		Reduction of the protrusive effect
Major and Minor of the Helix	Auricle	Auricle reshape
		Reduction of the auricle’s wrinkles
Tragus and Antitragus	Auricle	Armonization of the auricle with respect to the face

**Table 3:** BoNT-A selective neuromodulation treatment parameters of the Russo Ear Lift Technique.

Muscles	Superior Auricle Muscle	Posterior Auricle Muscle	Anterior Auricle Muscle	Helix Muscles
Inoculation Points	1 point in the superficial region above the ear, 1 cm from the insertion line	1 point in the posterior region of the muscle, 1.5 cm anterior to the mastoid process	1–2 points in the anterior region of the ear, 1 and 1.5 cm in front of the insertion on the temporalis fascia	External superior curvature of the auricle, 2–3 points at 0.5 cm from the margin of the helix
Inoculation Depth	Superficial/subcutaneous (to relax the muscle without weakening the support of the auricle)	Deep intramuscular, at a 45° angle to the skin	Superficial intramuscular (for a selective action without excessive diffusion)	Superficial/subcutaneous (to act on the skin folds without altering the cartilage structure)
Dosage	ONA: 1–2 U ABO: 3–5 U *	ONA: 2–4 U ABO: 5–10 U	ONA:1–2 U ABO: 3–5 U per point	ONA: 1 U ABO: 2–3 U per point
Effects	Reduction of traction on the upper auricle Improvement of wrinkles above the auricle Slight lowering of the ear, with natural projection	Reduction of ear protrusion, with improved projection Reduction of posterior tension that pulls the auricle forward Greater harmonization of facial profile	Reduction of anterior ear traction (excessive forward tilt avoided) Improvement of surrounding skin appearance Greater harmonization between auricle and zygomatic projection	Reduction of excessive curvature of helix Smoothing of skin folds (more loose appearance) Prevention of static wrinkles due to tension in the auricle
Possible Side Effects	Involvement of temporalis muscle if injection too profound and consequent chewing weakness	An excessively lateral injection may interfere with the mobility of the posterior cervical musculature	An injection too medial can affect eyebrow elevation	An overly superficial injection may cause a slight, transient local irritation

* U = units; for ONA, it refers to Allergan Units, while for ABO, it refers to Speywood Units.

## Data Availability

The data supporting the findings of this study are held by the specialist who performed the treatments at her center due to privacy reasons.

## Abbreviations

The following abbreviations are used in this manuscript:

ABO	AbobotulinumtoxinA
AEAC	Aesthetic Ear Aging Classification
BoNT	Botulinum Toxin
BoNT-A	Botulinum Toxin type A
CO2	Carbon dioxide
GAIS	Global Aesthetic Improvement Scale
HA	Hyaluronic Acid
ICC	Intraclass Correlation Coefficient
NSAIDs	Non-Steroidal Anti-Inflammatory Drugs
ONA	onabotulinumtoxinA
U	Units
